# Effect of whole-body vibration on reduction of bone loss and fall prevention in postmenopausal women: a meta-analysis and systematic review

**DOI:** 10.1186/s13018-016-0357-2

**Published:** 2016-02-17

**Authors:** Chiyuan Ma, An Liu, Miao Sun, Hanxiao Zhu, Haobo Wu

**Affiliations:** Department of Orthopaedic Surgery, Second Affiliated Hospital, School of Medicine, Zhejiang University, Hangzhou, China; Department of Oral and Maxillofacial Surgery, Second Affiliated Hospital, School of Medicine, Zhejiang University, Hangzhou, China

**Keywords:** Whole-body vibration, Bone mineral density, Fall, Meta-analysis, Systematic review

## Abstract

**Background:**

To examine whole-body vibration (WBV) effect on bone mineral density (BMD) and fall prevention in postmenopausal women, we performed a meta-analysis and systematic review of prospective randomized controlled trials (RCTs) comparing change in BMD of the femoral neck and lumbar spine and related factors of falls between WBV group and control group.

**Methods:**

EMBASE, PubMed, Cochrane Central Register of Controlled Trials, ISI Web of Science, and China National Knowledge Infrastructure (CNKI) were searched up to April 2015; search strategy was used as follows: (vibration) AND (osteoporo* OR muscle* OR bone mineral density OR BMD). All prospective randomized controlled trials comparing related factors of falls and BMD change in the femoral neck and lumbar spine between WBV group and control group were retrieved.

**Results:**

Eight of 3599 studies with 1014 patients were included, 477 in the WBV group, and 537 in the control group. We found that there was no significant difference in all magnitude groups of the femoral neck (*N* = 936, WMD: 0.00 (–0.00, 0.01); *p* = 0.18). A statistical significance showed in the all magnitude groups (*N* = 1014, WMD: 0.01 (0.00, 0.01); *p* = 0.01) and low-magnitude group (*N* = 838, WMD: 0.01 (0.00, 0.01); *p* = 0.007) of the lumbar spine. No significant difference was found in high-magnitude group of the lumbar spine (*N* = 176, WMD: 0.00 (−0.01, 0.02); *p* = 0.47), low-magnitude group (*N* = 838, WMD: 0.00 (−0.00, 0.00); *p* = 0.92) and high-magnitude group (*N* = 98, WMD: 0.02 (−0.00, 0.05); *p* = 0.06) of the femoral neck. All the studies provided data of related factors of falls such as strength of the lower limb, balance, and fall rate reported effectiveness of WBV therapy. In addition, no complication was reported.

**Conclusions:**

Low-magnitude whole-body vibration therapy can provide a significant improvement in reducing bone loss in the lumbar spine in postmenopausal women. Moreover, whole-body vibration can be used as an intervention for fall prevention.

## Background

Osteoporosis and osteoporotic fracture occurrence have been significant public health problems all around the world [1]. For the patients with hip fracture, one of the commonest osteoporotic-related fractures (hip fracture, lumber compressive fracture), the mortality is 12–20 % higher than other patients at similar age who have not suffered a fracture [2]. Among all patients suffering osteoporosis, the group of postmenopausal women constitutes a huge amount due to dramatic decrease of estrogen, which plays an important role in female bone loss [3]. Treatment which could increase bone mass or decelerate the loss of bone after menopause may result in a lower occurrence of osteoporotic fracture for postmenopausal women [4]. The traditional pharmacologic intervention contains a series of drugs, such as teriparatide and bisphosphonate, of which the long-term safety still remains unknown [5–7].

Whole-body vibration (WBV) is a new promising anti-osteoporotic treatment in the postmenopausal women. The vibration is transmitted to the patient through a vibration platform where she stands. The intensity of WBV is defined by its frequency (hertz) and magnitude (g, 1g = 9.8 m/s^2^). Mechanical signals introduced via vibration have been shown to stimulate bone formation [8]. Animal studies also showed the effectiveness of WBV in increasing bone mass and improving bone architecture and strength [9, 10]. Also, some clinical trials have indicated that WBV can benefit to bone mineral density (BMD) change in postmenopausal women [11–13]. However, not all of studies came to the same conclusion [14, 15], which showed no significant improvement in the WBV group. One systematic review in 2010 [16] made a data pool of change in BMD from five randomized controlled trials and demonstrated that WBV could provide small but significant improvements in BMD of the hip area in postmenopausal women. In addition, this kind of new intervention could improve balance or strength of patients which would be related to the risk of fall or osteoporotic fracture.

To evaluate the musculoskeletal effect of whole-body vibration (WBV) in postmenopausal women, we performed a systematic review and meta-analysis of the RCTs comparing the change in BMD and related factor of falls in postmenopausal women.

## Methods

We strictly follow the methods established in the Cochrane Handbook for Systematic Reviews of Interventions 5.0.2 and the PRISMA 2009 checklist [17].

### Literature search

Three reviewers (CM, AL, HZ) searched electronic database (EMBASE, PubMed, Cochrane Central Register of Controlled Trials, ISI Web of Science, and China National Knowledge Infrastructure (CNKI) without limit independently. Results were last updated on April 6, 2015. Search strategy was used as follows: (vibration) AND (osteoporo* OR muscle* OR bone mineral density OR BMD) without limitation of publication year or language. In order to detect other reports not get by our original search, we also hand-searched the reference lists of manuscripts included. The reviewers also inquired experts in the area of WBV or osteoporosis to get unpublished trials. The titles and abstracts were reviewed by two reviewers following the standards: (1) evaluation of the effects of WBV on BMD in postmenopausal women; (2) only WBV performed with standing body is included; (3) as it takes at least half a year for BMD to show a significant response, the follow-up period should be more than 6 months; (4) the patient in the trials should not have any other disease that could influence the BMD; and (5) prospective randomized controlled trial. Exclusion criteria include (1) retrospective studies, observational studies, case reports, or reviews; (2) WBV performed with lying body, as lying one is through different mechanism. Otherwise, it would affect the result of analysis [18]; (3) cadaveric research; (4) no available outcome data; and (5) the follow-up time was under the standard. The redundant publications were excluded by title review. Then, the abstracts of the remaining studies were reviewed to meet the above criteria. At last, the full texts were read in detail. All eligible trials met inclusion criteria exactly.

### Data extraction

Two investigators (CM, MS) extracted data from included studies. Especially, study design, patient demographics (sample size, age), WBV therapy, mean follow-up time, calcium and vitamin D requirements, loss to follow-up rate, change of BMD of the femoral neck and lumbar spine, and related factors of falls were abstracted. WBV was defined as mechanical vibration, performed with a straight body. Local body vibration or ultrasound was not regarded as WBV. The data of WBV therapy included frequency, magnitude, prescribed, and actual mean cumulative volume (total number of minutes per study). Related factors of falls included fall rate, data of balance, and leg strength. Intention-to-treat (ITT) data from the trials was used. If the relevant data were not reported in the article, we tried to get them from the accompanying graphs. We also tried to get in touch with the authors of the eligible trials to get further data if needed.

### Quality assessment

Two investigators (AL, HW) independently assessed the methodological quality of each study according to the 12-item scale [19]: randomized adequately, allocation concealed, similar baseline, patient blinded, care provider blinded, outcome assessor blinded, avoided selective reporting, similar or avoided cofactor, patient compliance, acceptable drop-out rate, similar timing, and intention-to-treat (ITT) analysis. Kappa test was used to assess the divergences, and consensus was obtained by the discussion with the third investigator. According to the 12-item standard (Table 1), five high-quality [13–15, 20, 21] studies explicitly introduced the randomization and the allocation concealment and described ITT analysis; the other three studies [11, 12, 22] received moderate quality. The weighted kappa for the agreement on the study quality assessment between the reviewers was 0.88 (95 % confidence interval (CI), 0.82–0.94).

**Table 1:** Methodological quality of the included studies based on the 12-item scoring system

Study	Randomized adequately^a^	Allocation concealed	Patient blinded	Care provider blinded	Outcome assessor blinded	Acceptable dropout rate^b^	ITT analysis^c^	Avoided selective reporting	Similar baseline	Similar or avoided cofactor	Patient compliance^d^	Similar timing	Quality
Verschueren et al., 2004 [11]	Yes	Yes	Yes	No	Yes	Yes	No	Yes	Yes	Yes	Yes	Yes	Moderate
Rubin et al., 2004 [20]	Yes	Yes	Yes	No	Yes	Yes	Yes	Yes	Yes	Yes	Yes	Yes	High
Iwamoto et al., 2005 [22]	Yes	No	Yes	No	Yes	Yes	Yes	Yes	Yes	Yes	Yes	Yes	Moderate
Gusi et al., 2006 [12]	Yes	No	Yes	No	Yes	Yes	No	Yes	Yes	Yes	Yes	Yes	Moderate
Stengel et al., 2011 [14]	Yes	Yes	Yes	No	Yes	Yes	Yes	Yes	Yes	Yes	Yes	Yes	High
Stengel et al., 2011 [15]	Yes	Yes	Yes	No	Yes	Yes	Yes	Yes	Yes	Yes	Yes	Yes	High
Lai et al.,2013 [13]	Yes	Yes	Yes	No	Yes	Yes	Yes	Yes	Yes	Yes	Yes	Yes	High
Leung et al., 2014 [21]	Yes	Yes	Yes	No	Yes	No	Yes	Yes	Yes	Yes	No	Yes	High

^a^Only if the method of sequence made was explicitly introduced could get a “Yes”; sequence generated by “Dates of Admission” or “Patients Number” receive a “No”

^b^Dropout rate <20 % could get a “Yes”, otherwise “No”

^c^ITT = intention-to-treat, only if all randomized participants were analyzed in the group, they were allocated to receive a “Yes”

^d^More than 75 % patients accept respective treatment for at least 6 weeks means “Yes”, otherwise “No”

### Statistical analysis

All data were conducted with Review Manager (RevMan) [Computer program] (Version 5.3. Copenhagen: The Nordic Cochrane Centre, The Cochrane Collaboration, 2014). We used relative risk (RR) and 95 % CI for the analysis of dichotomous outcomes. Standardized mean difference (SMD) or weighted mean difference (WMD) was calculated with 95 % CI as the summary statistics for continuous data. We used a chi-squared test on N-1 degrees of freedom to evaluate the statistical heterogeneity, with significance at 0.05. *I*^2^ (*I*^2^ = ((*Q* − *df*)/*Q*) × 100 %) was used to calculate the percentage of the variability in effect estimates according to the heterogeneity. *Q* means the *x*^2^ statistic and *df* is the degree of freedom. A chi-squared test and *I*^2^ test were used to calculate the statistical heterogeneity. We considered *I*^2^ values of 25, 50, and 75 % as low, medium, and high heterogeneity, respectively. If *I*^2^ < 50 %, the fixed effects model was used; otherwise, we used the random effects model. We conducted sensitivity analyses through omitting trials to figure out the source of high heterogeneity and to evaluate whether specified factors (methodological parameters: ITT analysis, adequate randomization; potential relevant modifiers: control design, WBV therapy) could influence the total effects of BMD change and fall factors. We conducted such sensitivity analyses when there were three or more trials included in the comparison. We aimed at the magnitude as a major difference of WBV therapy. In order to compare with the previous meta-analysis, we also divided the RCTs into low-magnitude group (magnitude <1 g) and high-magnitude group (magnitude ≥1 g) as subgroup analysis [16]. The Grading of Recommendations Assessment, Development and Evaluation (GRADE) approach was also applied to each analysis performed to evaluate the quality of evidence [23].

## Results

### Literature review

**Fig. 1:**
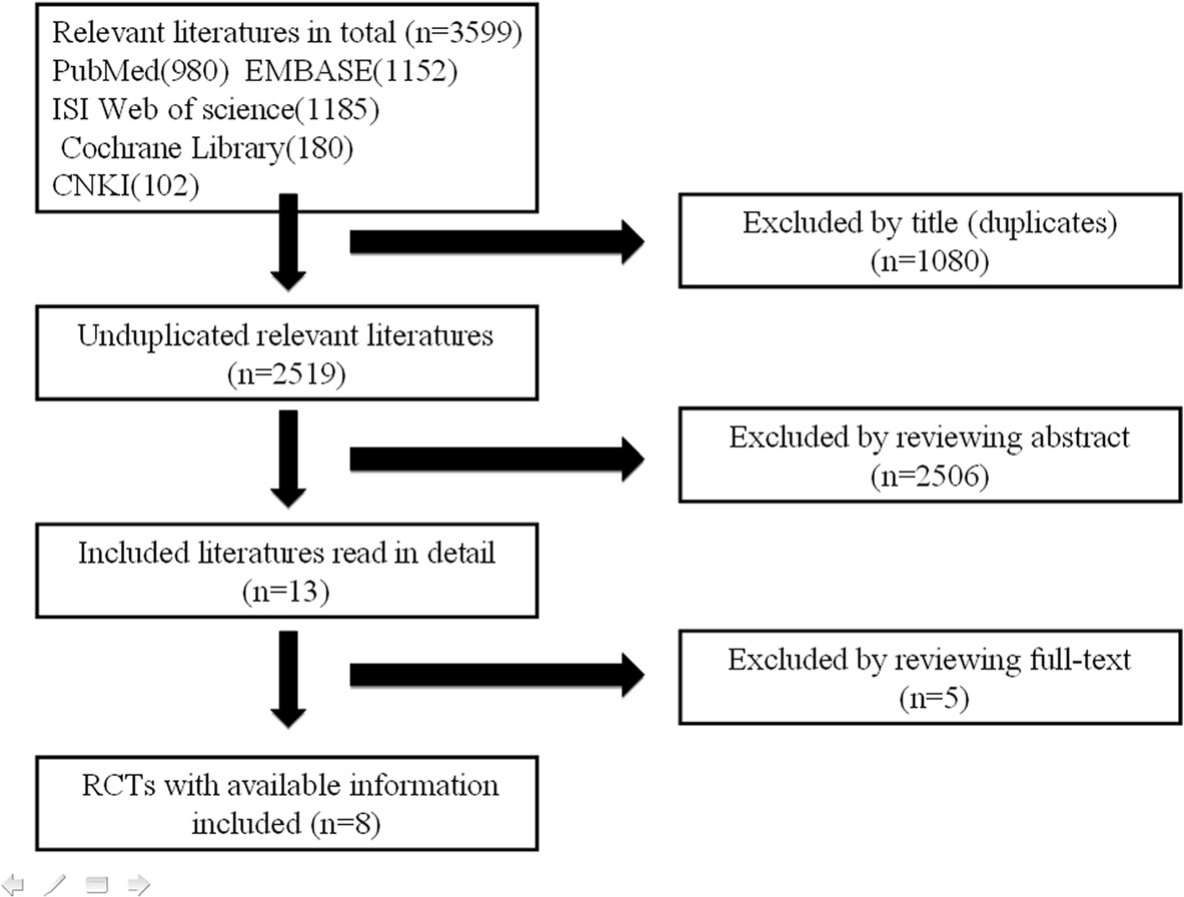
A PRISMA flowchart illustrated the selection of studies included in our systematic review

Literature search initially found 3599 relevant citations. Among them, there were 1080 duplicates leaving 2519 trials. After reviewing titles and abstracts according to the eligible criteria, only 13 were retrieved in full text. Two of them were not controlled trials; three lacked any clinical follow-up data of more than 6 months. Finally, eight prospective randomized controlled trials met eligibility criteria (Fig. 1). The weighted kappa for the agreement on eligibility between the investigators was 0.86 (95 % CI, 0.78–0.96).

### Characteristics and interventions

The characteristics and interventions of eight trials are presented in Table 2. They were all prospective randomized controlled trials. One thousand fourteen patients were included, 477 in the WBV group, and 537 in the control group. The frequency and magnitude of WBV therapy were described in all trials. Five of eight included studies treated the patients with calcium [12, 14, 15, 20, 22]. In three of them [12, 14, 15], vitamin D was also given to the patients. The minimum length of follow-up was more than 6 months. Dropout rate (≤20 %) was acceptable in seven of eight trials [11–15, 20, 22]. According to the magnitude, we divided the RCTs into low-magnitude group [14, 15, 20, 21] (magnitude <1 g) and high-magnitude group [11–13, 22] (magnitude ≥1 g).

**Table 2:** Study characteristics

Study	Age (years)	Sample size (WBV/CON)	WBV therapy			Control intervention	Calcium requirements	Vitamin D requirements	Mean follow-up (months)	Loss to follow-up rate (%)
			Frequency (hertz)	Magnitude (g)	Mean cumulative volume (minutes)					
Verschueren et al., 2004 [11]	58–74	(25/45)	35–40	≥1	1021	No treatment and resistance training	None	None	6	0
Rubin et al., 2004 [20]	47–64	(33/37)	30	<1	5840	Sham vibration	Measured intake	None	12	20
Iwamoto et al., 2005 [22]	55–88	(25/25)	20	≥1	208	No treatment	>800 mg through diet per day	None	12	0
Gusi et al., 2006 [12]	66 ± 5	(14/14)	12.6	≥1	494	Walking	Measured intake	Measured intake	8	0
Stengel et al., 2011 [14]	65.8 ± 3.5	(36/36)	35	<1	2340	Sham vibration	Measured intake <1200 mg per day	Measured intake <800 IU	12	7
Stengel et al., 2011 [15]	68.5 ± 3.1	(50/50)	25–35	<1	2340	Conventional training group	1500 mg per day	400 IU per day	18	7
Lai et al.,2013 [13]	69.5 ± 2.25	(14/14)	30	≥1	468	No treatment	None	None	6	0
Leung et al.,2014 [21]	73 ± 7.0	(280/316)	35	<1	7200	No treatment	None	None	18	22.3

### BMD

**Fig. 2:**
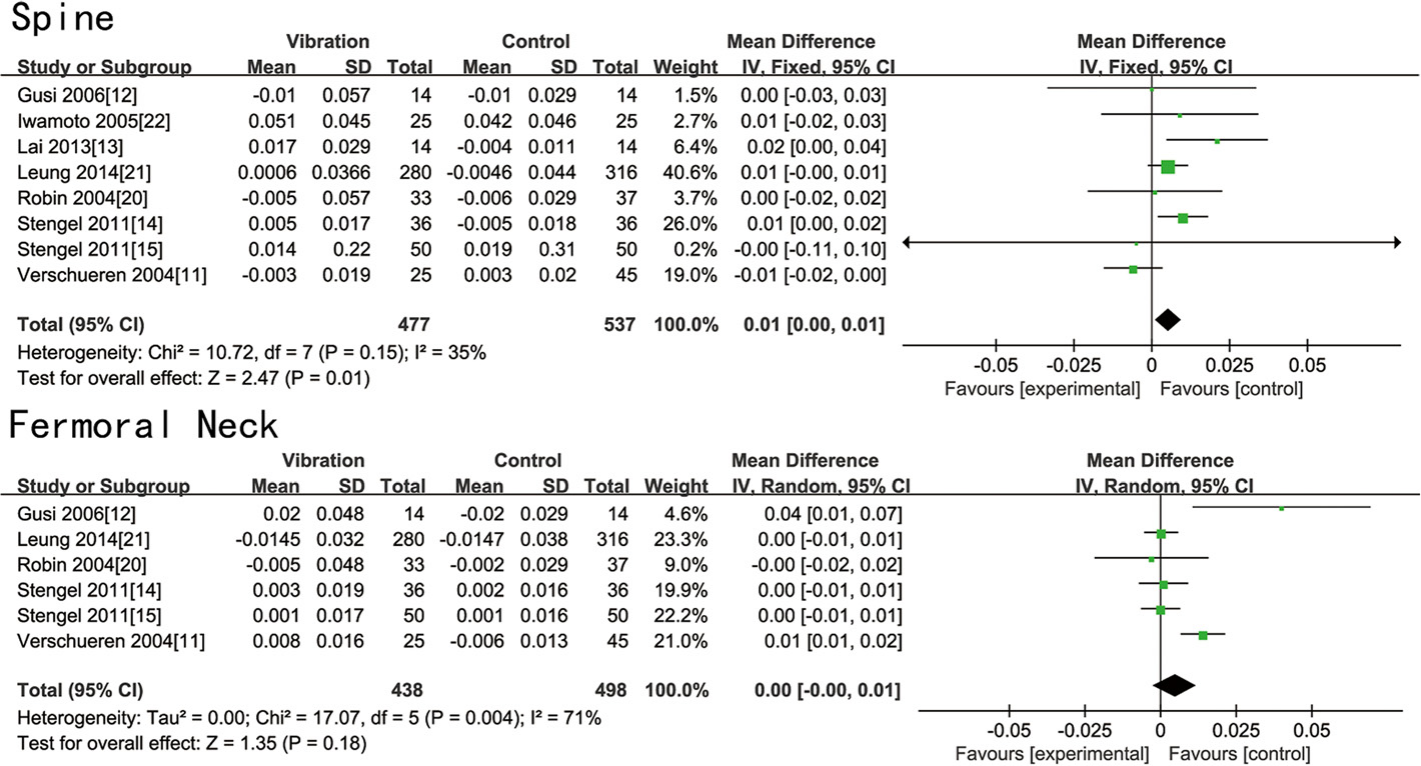
Forest plots for the BMD change of the lumbar spine and femoral neck between the WBV and CON group in all magnitude groups

**Fig. 3:**
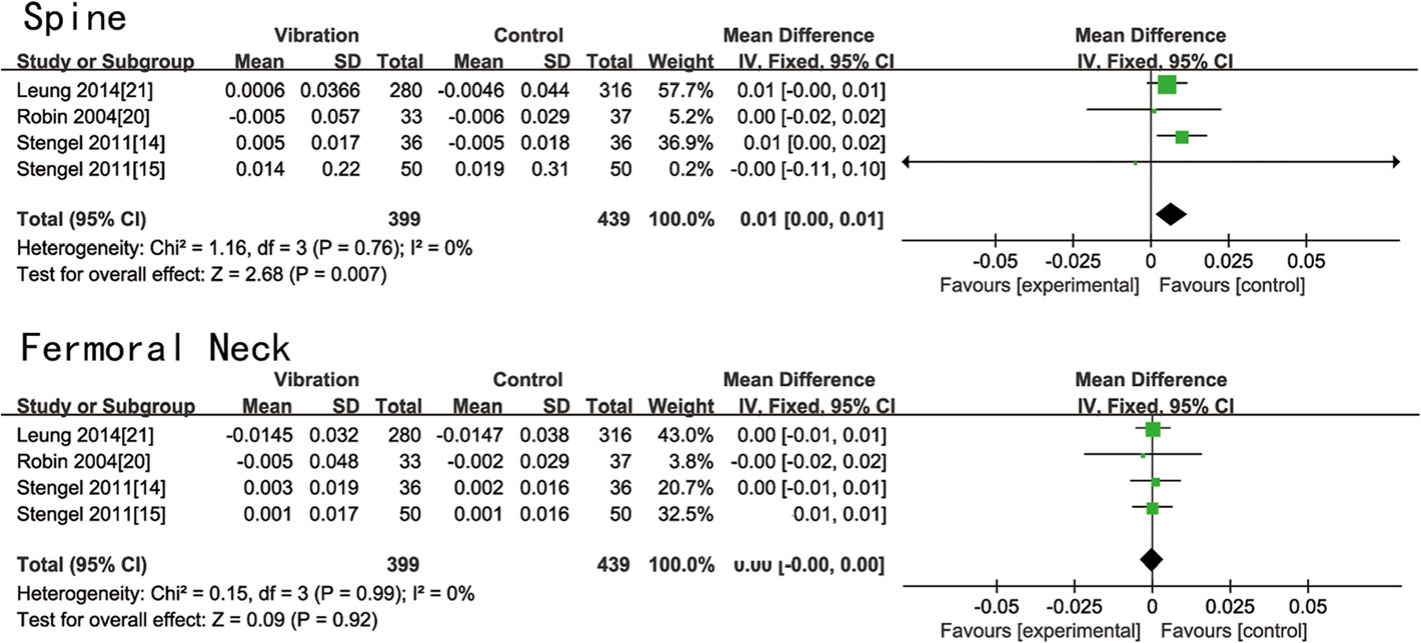
Forest plots for the BMD change of the lumbar spine and femoral neck between the WBV and CON group in low-magnitude group

**Fig. 4:**
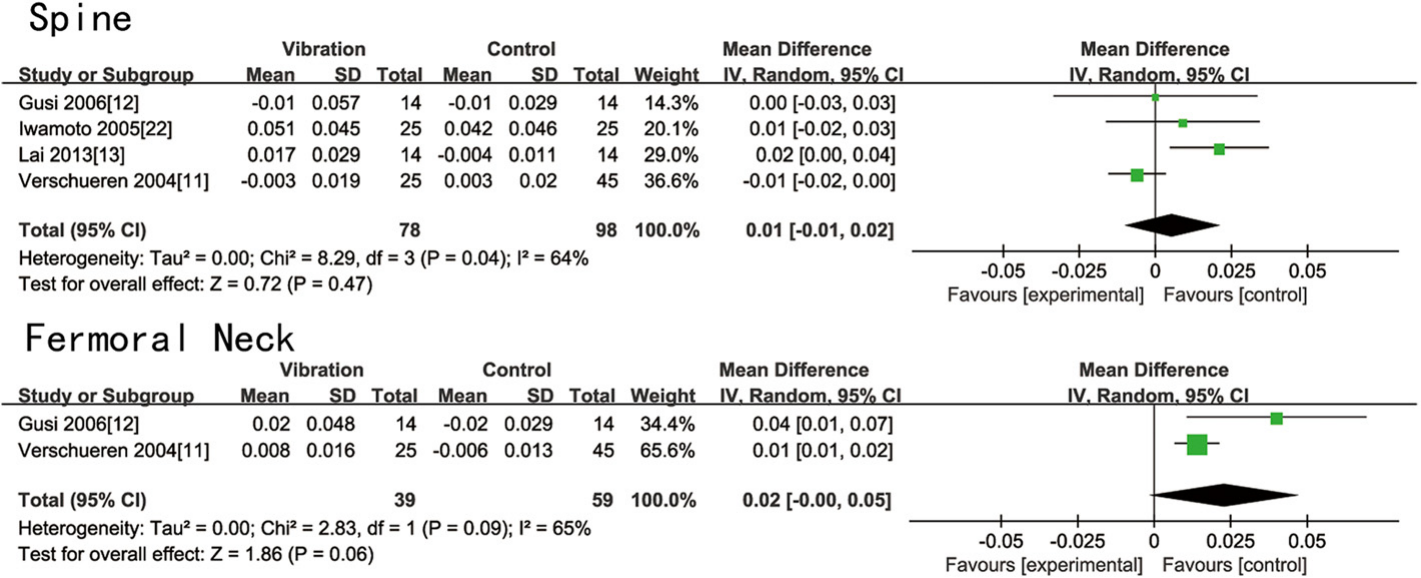
Forest plots for the BMD change of the lumbar spine and femoral neck between the WBV and CON group in high-magnitude group

All the included trials presented the change in BMD of the lumbar spine in postmenopausal women at baseline and after treatment. Six of them provided the data of BMD change of the femoral neck. Table 3 displayed all the data of BMD change of the femoral neck and lumbar spine. According to Table 3, WBV is effective in reducing bone loss at the lumbar spine in two trials [13, 14], while the other six [11, 12, 15, 20–22] showed no statistical significance. WBV is beneficial to the BMD of the femoral neck in two trials [11, 12], while no difference between two groups in the other four trials [14, 15, 20, 21]. All data were pooled to make a meta-analysis. We found that there was no significant difference in all magnitude groups of the femoral neck (*N* = 936, WMD: 0.00 (−0.00, 0.01); *p* = 0.18) (Fig. 2). A statistical significance showed in the all magnitude groups (*N* = 1014, WMD: 0.01 (0.00, 0.01); *p* = 0.01) (Fig. 2) and low-magnitude group (*N* = 838, WMD: 0.01 (0.00, 0.01); *p* = 0.007) (Fig. 3) of the lumbar spine. We find no significant difference in high-magnitude group of the lumbar spine (*N* = 176, WMD: 0.00 (−0.01, 0.02); *p* = 0.47) (Fig. 4), low-magnitude group (*N* = 838, WMD: 0.00 (−0.00, 0.00); *p* = 0.92) (Fig. 3) and high-magnitude group (*N* = 98, WMD: 0.02 (−0.00, 0.05); *p* = 0.06) (Fig. 4) of the femoral neck. Low heterogeneity was shown in low-magnitude group of the femoral neck (*I*^2^ = 0 %), all magnitude groups (*I*^2^ = 35 %) and low-magnitude group (*I*^2^ = 0 %) of the lumbar spine. Medium heterogeneity was shown in high-magnitude group of the lumbar spine (*I*^2^ = 64 %), all magnitude groups (*I*^2^ = 71 %), and high-magnitude group (*I*^2^ = 65 %) of the femoral neck. After omitting moderate-quality studies, we found significant difference in all magnitude groups of the BMD change of the lumbar spine (*N* = 270, WMD: 0.01 (0.00, 0.02); *p* = 0.002) with decreased heterogeneity (*I*^2^ = 0 %).

**Table 3:** Change in BMD and fall-related factors

Study	Group	Absolute pre-post change in BMD (mean ± SD)		Fall-related factors
		Femur neck (g cm^−2^)	Spine (g cm^−2^)	
Verschueren et al., 2004 [11]	WBV	0.008 ± 0.016	−0.003 ± 0.019	Change of knee extensor isometric strength (N m): 18.3 ± 22.95
	CON	−0.006 ± 0.013	0.003 ± 0.020	Change of knee extensor isometric strength (N m): 6.34 ± 23.81
Rubin et al., 2004 [20]	WBV	−0.005 ± 0.048	−0.005 ± 0.057	N.A.
	CON	−0.002 ± 0.029	−0.006 ± 0.029	
Iwamoto et al., 2005 [22]	WBV	N.A.	0.051 ± 0.045	N.A.
	CON		0.042 ± 0.046	
Gusi et al., 2006 [12]	WBV	0.020 ± 0.048	−0.010 ± 0.057	Balance change (trials): −2.7 (95 % CI, −5.7 to −0.1)
	CON	−0.020 ± 0.029	−0.01 ± 0.029	Balance change (trials): 0.5 (95 % CI, −0.9 to 0.6)
Stengel et al., 2011 [14]	WBV	0.003 ± 0.019	0.005 ± 0.017	Change of leg extension isometric strength (N): 166 ± 144.4
	CON	0.002 ± 0.016	−0.005 ± 0.018	Change of leg extension isometric strength (N): 37.1 ± 129.9
Stengel et al., 2011 [15]	WBV	0.001 ± 0.017	0.014 ± 0.22	Fall rate (falls/person): 0.70 ± 0.83
	CON	0.001 ± 0.016	0.019 ± 0.31	Fall rate (falls/person): 0.96 ± 1.10
Lai et al.,2013 [13]	WBV	N.A.	0.017 ± 0.029	N.A.
	CON		−0.004 ± 0.011	
Leung et al., 2014 [21]	WBV	−0.0145 ± 0.032	0.0006 ± 0.0366	Adjusted hazard ratio of fall or fracture (95 % CI): 0.56 (0.40, 0.78)
	CON	−0.0147 ± 0.038	−0.0046 ± 0.044	

*N.A.* not available

### Related factors of falls

Five studies [11, 12, 14, 15, 21] included related factors of falls (Table 3). Verschueren et al. [11] performed a maximal voluntary isometric contraction of the knee extensors to assess the muscle strength at baseline and after 6 months, which demonstrated an improvement of isometric strength in WBV group with significant difference (*p* < 0.001). Gusi et al. [12] assessed the postural balance at baseline and 8 months with a blind flamingo test, which showed a significant improvement of balance in WBV group (*p* = 0.023). Stengel et al. [14] demonstrated a significant improvement of leg strength in WBV group by measuring maximum isometric leg extension strength (*p* < 0.001). Stengel et al. [15] showed a significant difference between the fall rates in 18 months of two groups (*p* = 0.003). Leung et al. [21] found the improvement of fall or fracture rate of WBV group in a cluster-randomized controlled trial. As different related factors of falls were used in all five studies, data synthesis was inappropriate.

### GRADE analysis

GRADE analysis showed comprehensively moderate quality in the outcomes of the pre-post change in BMD in low-magnitude group of the femoral neck, all magnitude groups, and low-magnitude group of the lumbar spine. It resulted from inadequate blinding and lack of concealed allocation. As the obvious heterogeneity had a negative effect on the quality, the quality of the evidence was low for the pre-post change in BMD in high-magnitude group of the lumbar spine, all magnitude groups, and high-magnitude group of femoral neck.

## Discussion

Physical exercises are believed to be effective in reducing bone loss and fall rate [24–26]. However, the compliance of long-term physical exercises is poor, and sometimes, it may increase the risk of injures, [27] especially in elderly individuals. With better compliance and safety to postmenopausal women, WBV is regarded as a new potential anti-osteoporotic treatment. However, its positive effect on bone quality improvement and fall prevention has not been confirmed yet.

The systematic review [16] in 2010 demonstrated that WBV could provide small but significant improvements in BMD of the hip area in postmenopausal women. However, per-protocol data was used for meta-analysis and only five RCTs were available, which would weaken the level of evidence.

According to the result of our analysis, there is no statistical difference in change in BMD of the femoral neck between two groups for all magnitude, low-magnitude, or high-magnitude WBV. However, the analysis of low-magnitude and all magnitude WBVs found significant improvement in lumbar spine BMD change in postmenopausal women, with no significant difference in high-magnitude WBV. The difference of effectiveness between high-magnitude and low-magnitude WBVs may have something to do with the transmission mechanism of vibration in human body. However, to the best of our knowledge, no such existing relevant study has been reported.

According to Wolff’s law of bone remodeling, [28] only large-magnitude strains can construct new bone, so the greater the magnitude is, the greater the effect should be. However, animal studies [9, 10, 29, 30] suggested that low-magnitude vibration could also enhance bone accrual. Thus, based on the magnitudes, we stratified included RCTs into two subgroups for meta-analysis and achieved the similar results like the experimental animal models.

The commonest direct reason of osteoporotic fracture is fall with poor bone condition in postmenopausal women. Falls can lead to functional decline and fragility of bone [31]. To the best of our knowledge, our analysis is the first systematic review to extract the data of related factor of falls to evaluate the risk of fall after WBV treatment in postmenopausal women. Factors related to falls are various including balance disfunction, muscle weakness, impaired gait, and mobility [32, 33]. Although different factors were evaluated in the included studies [11, 12, 14, 15], all the studies demonstrated improvement of related factors of falls of WBV group including isometric strength, balance, and fall rate.

Changes in blood flow may indicate a possible mechanism of WBV treatment. Blood flow and muscle oxygenation are closely related. Blood flow to the muscle in response to the increased demands for oxygen and increased carbon dioxide and hydrogen ion concentrations during physical exercises. With the use of near-infrared spectroscopy (NIRS), several studies [34–36] suggested that WBV could increase peripheral blood flow. Ischemia can cause decreased availability of energy because oxygen serves as the final electron acceptor in the electron transport chain [37]. On the contrary, balance function and muscle strength is enhanced with the improvement of blood supply resulting from the WBV therapy.

### Study limitations

(1) The variations of the control group of the included studies. Three studies [13, 21, 22] provided no treatment to the patients in the control group. Sham vibration was used in two included trials [14, 20]. The control group in the other three trials [11, 12, 15] received physical exercise training. This differentiation could have a negative impact on the level of evidence. (2) In addition to whole-body vibration therapy, anti-osteoporosis drugs such as calcium, vitamin D, and alendronate were used in several included studies [12, 14, 15, 20, 22]. Although there is no between-group difference of anti-osteoporosis drug use, it is possible that anti-osteoporosis drug therapy plus WBV treatment confers a greater than addictive effect. These two therapies may enhance the effect of each other. (3) Short follow-up time. All included trials provided follow-up data of no longer than 18 months, and no complication was reported. However, long-term effect of WBV still needs to be evaluated. (4) Lack of systematic unified fall risk assessment. To the best of our knowledge, there’s no such an assessment that can be used to evaluate the fall risk. Different assessment or test was used in included studies, which makes it inappropriate to do meta-analysis. Thus, a systematic assessment should be formulated to evaluate the fall risk in postmenopausal women. Such assessment should synthesize the data of strength of lower limbs, results of balance test, BMD, etc. and come to a score as result.

## Conclusions

Low-magnitude whole-body vibration therapy can provide a significant improvement in reducing bone loss in the lumbar spine in postmenopausal women. Moreover, whole-body vibration can be used as an intervention for fall prevention.

## Abbreviation

BMD: bone mineral density
WBV: whole-body vibration
